# Art and mentalizing in early intervention psychosis: rationale and therapist insights on a new psychoeducational course

**DOI:** 10.3389/fpsyt.2024.1243990

**Published:** 2024-07-01

**Authors:** Sarah Parkinson, Bethany Cole, Clare Trevelyan

**Affiliations:** Avon and Wiltshire Mental Health Partnership National Health Service (NHS) Trust, Bath, United Kingdom

**Keywords:** art therapy, mentalizing, first episode psychosis, early intervention, psychoeducation, joint attention, MBT, attachment

## Abstract

In this paper, we explore the rationale for a combined art therapy and mentalization-based treatment (MBT) group course for those experiencing a first episode of psychosis (FEP). We discuss the theoretical background for how art and MBT theory can help us better understand and work with groups of individuals experiencing FEP, particularly focusing on avoidance and insecure attachment styles. We outline the delivery of a ten-week psychoeducational Art MBT course within an Early Intervention in Psychosis (EIP) Service and discuss our experiential insights into this new modality as co-therapists. We conclude by proposing that art therapy and mentalizing practice together offer an accessible, useful and practical group structure for EIP services, which could improve individuals’ mentalizing capacity and overall social functioning.

## 1 Introduction

Art psychotherapy is included in United Kingdom (UK) national guidelines for psychosis care and treatment (1). Pilot randomised controlled trials on art therapy in psychosis have demonstrated improvements in social and emotional awareness (2), negative symptoms (3) and changes in attitudes to self and others (4). A major trial, ‘MATISSE’ however, suggested that art therapy did not offer benefits over and above usual care (5). Criticism of this trial identified a lack of clarity about the format and content of art therapy practice in relation to psychosis (6, 7). A subsequent literature review concluded that therapists and patients consider art therapy beneficial, meaningful and acceptable for psychosis, but again suggested that practice was in need of clearer and more consistent definition (8).

In 2013, an art therapy group was piloted in our local Early Intervention in Psychosis (EIP) service in the UK National Health Service (NHS) (9), basing its design on both national (1) and EIP guidelines (10). Individuals attending this group highlighted the importance of peer support, working in a group, and not feeling under pressure in therapy. The pilot explored how to find a balance that would help understand and address issues of avoidance while not putting individuals under too much pressure. Increasing the session structure was identified as potentially helpful in guiding group practice, with the recommendation that adopting a mentalizing approach may help individuals maintain the focus on experiences and emotions that may be otherwise avoided. Alongside attending to avoidance, we propose that combining art therapy and mentalizing practice may enhance feelings of trust with others, develop shared attention and enhance mentalizing capacity. This may enable the activity of social learning and increased social functioning; one of the three key areas to attend to in EIP services, alongside the psychological impact of trauma and biological impact of distress (10).

Mentalization describes a “*profoundly social construct in the sense that we are attentive to the mental states of those we are with, physically or psychologically*”, a process that is both imaginative and helps us make sense of each other and ourselves (11). Specific impairments in mentalizing and ‘Theory of Mind’ tasks in adults with psychosis are well established (12, 13). Debbané proposes that those with psychosis can feel disconnected from their sensory and mental functions with a profound loss of agency over their thoughts, feelings and behaviours (14). This feeling of alienation from the self and the external reality can lead to psychotic symptoms, such as hallucinations. Disturbances in experiencing self and reality can be highly distressing and are potentially underappreciated by clinicians (15).

Debbané proposes that these core self-disturbances are associated with a sustained cycle of impaired mentalizing, where pre-mentalizing modes can take over to rescue agency over selfhood (14). Pre-mentalizing modes refer to modes of functioning that help people with disturbed mentalizing organize their experience of themselves and others. There are three basic modes: ‘Psychic equivalence’ or ‘inside-out’ thinking (16) is a state in which inner thoughts and feelings become ‘too real’ and the individual experiences their own, often frightening thoughts and feelings, reflected back from the world around them with no adjustment or understanding. For example, an individual might initially develop a fixed paranoid delusional belief from worries about how others’ think of them. ‘Teleological mode’ is recognised when mental states are expressed or understood in terms of actions alone, as if emotional difficulties can only be resolved in ‘doing’. An example of this might be individuals misunderstanding ordinary events or attributing undue significance to them (for example, *if the traffic light turns red, I will turn left at the junction, this must be a message from God*). ‘Pretend mode’ describes a state in which thoughts and feelings become disconnected from reality; the obvious (‘elephant in the room’) is overlooked. An individual may talk verbosely or tangentially without engaging others in genuinely understanding them.

Attachment theory suggests that early relationships between children and their caregivers shape the individual’s subsequent interpretations and expectations of relationships (17). Insecure attachment styles contribute to these disturbances in mentalizing and also to the development of epistemic mistrust (18). Epistemic trust relates to social learning and the ability to learn from others, particularly in how to navigate a complex social world (14). In contrast, epistemic mistrust results in resistance to other people’s views, particularly when their views conflict with previously held beliefs. This mistrust cuts the individual off from social learning and the opportunity to update belief systems (18). Understanding of both one’s own and other’s mental states develops in early attachment relationships involving caregivers and child. However, when there is emotional misattunement within that relationship, insecure attachment styles can develop (17, 19). For some, this can lead to exaggerated claims of self-sufficiency and pretence of independence and may manifest clinically in avoidance of close relationships (20). A high proportion of individuals living with psychosis show evidence of insecure attachment styles (21); a meta-analysis showed that 76% of individuals with psychosis had insecure attachment styles, in comparison to 38% of non-clinical groups (22). Furthermore, Carr et al. found that a ‘fearful’ (often described as disorganised) attachment style was most prevalent, thought to arise from experiencing either disrupted care experiences (such as neglect or loss) or frightening behaviour (such as abuse) during childhood (22). Adults with fearful attachment styles can present as highly anxious and avoidant due to conflicting feelings around both needing and resisting emotional closeness (23). Mentalization-based treatment’s (MBT) roots in attachment theory maintain the focus on social relatedness; indeed, focusing more on the therapeutic relationship than other more widely available therapies, such as Cognitive Behavioural Therapy for psychosis.

Addressing deficits in mentalizing are vital to recovery from psychosis, as they are one of the strongest predictors of functional outcome (24). Impaired mental state attribution is the single best predictor of poor social competence in schizophrenia (25). Mentalizing-based approaches are also effective in building resilience for people vulnerable to psychotic experience (16) and potentially improving social functioning in those who transition to non-affective psychosis, especially individuals with recent-onset psychosis (18). In terms of social functioning, MBT for psychosis is best provided sooner rather than later (16, 26), including for individuals ‘at risk’ of psychosis, or Clinical High Risk States, echoing the known value of an ‘early intervention’ approach (10).

Evidence for combined MBT and art psychotherapy practice is well established for those with a diagnosis of Personality Disorder (PD) (27–32). Similar evidence specifically regarding psychosis is limited and mostly focuses on changes in mentalizing capacity as a result of adapted psychodynamic art therapy practice, including a study suggesting significant shifts in the use of reflective functioning (the capacity to understand ourselves and others in terms of intentional mental states) through the use of art (33).

Building on the advances of MBT in understanding the role of the attachment system as a mechanism of change, Springham explores how mentalizing might operate within the use of art-making in therapy (34). As distinct from verbal psychotherapies, the basic relationship in art therapy is triangular: between two humans and an art object. The art objects are physically made, with the making process detectably preserved in their structure. They remain over time and are subject to joint viewing by individuals. As such, the art objects carry particular qualities that extend the possibilities of how individuals interact with those around them, both verbally and non-verbally (31, 34, 35).

Art making in therapy can be used to test out an idea or feeling in advance of it being made explicit (28, 36). It is seen to promote mentalizing by allowing the internal to be expressed externally and verbalised at a distance (36). Explicit and external mentalizing can be understood by looking at the MBT theory of ‘dimensions’ (37). Dimensions are described as social-cognitive activities, which represent the movement between two poles that occurs in response to changes both inside (mental states) and outside (the social environment) of ourselves. The four dimensions include ‘automatic versus (v) controlled’ (also referred to in this paper as implicit v explicit) to describe the shift between ‘moving along without thinking’ i.e. automatically, and pausing to ‘think twice’ or in a more controlled or explicit way. In art therapy, this can be used to describe the movement between absorption in drawing and making the idea explicit by putting words to the process. ‘Self v other’ refers to the shift between self-mentalizing and mentalizing the other. ‘Cognitive v affective’ describes the ability to identify and use reasoning in regard to mental states and at the other pole to be concerned with feeling. ‘Internal v external’ mentalizing refers to thinking about mental states in terms of imagining what might be going on inside oneself or another person, as distinct from picking up on external cues such as facial expression.

The opportunity to anchor mental content in an external form (the art object) may help to slow down the process of mentalizing to a manageable pace (30), maintain stability and avoid pre-mentalizing modes. The process of art-making necessitates multiple shifts between internal v external and implicit v explicit dimensions, allowing thoughts and feelings to be converted into words over time (28, 30, 36). These shifts can be seen in the to and fro between self-reflection in art making and interpersonal reflection in art sharing (30). The process of ‘anchoring’ mental content in art work is understood to hold some of the emotion (38), allowing an individual space to think and restore the cognitive v affective balance needed to mentalize (30). In addition, the created art objects may help clarify ownership of mental content, addressing self v other confusion. Lastly, the act of ‘looking together’ at each other’s work in art therapy offers a model of joint attention, described as the sharing of attentional focus and affect around a common object (39, 40). This allows individuals to contrast their perception of themselves (and their artwork) with how others perceive them (37, 41).

## 2 The Art MBT course

Below we outline the structure of the Art MBT course, including adaptions for those experiencing a First Episode of Psychosis (FEP). Following this, we discuss three therapists’ experiential insights and outline recommendations for future practice and research.

The Art MBT course for FEP was piloted in 2016 in an NHS EIP service in a predominantly rural area of the UK. The course was offered to all individuals under the local EIP service, run by an Art Psychotherapist and a Psychiatric Registrar. The course was held in a local community art studio, moving away from the clinical setting to reduce stigma and encourage active involvement in community based ventures (10, 42, 43). Five individuals, three males and two females, aged 21 to 30, completed this first course.

The purpose of the Art MBT course was to introduce an understanding of attachment theory within an art therapy and MBT framework. Additionally we wanted to ground the work in a shared understanding of art therapy, mentalizing and psychosis. We adopted a psychoeducational format to help us present theories and facts in a straightforward way giving group members and ourselves a shared language. We hoped this would help open up the conversation, using both art-making and words, about individual experience. The ten-week course was based on the MBT Introductory programme for Borderline PD (a twelve session psychoeducational module) (37) and covered an introduction to mentalizing, basic emotions, attachment, mentalizing culture, anxiety, trauma and depression. Differences with the PD course included the addition of a module on compassion (44) and a more in-depth exploration of mentalizing cultures [an everyday culture or group e.g. a family group or MBT group that discusses why people behave the way they do, in an open minded way (37)]. The mentalizing culture session was held in a local museum/art gallery, with the intention of making further connections to the social and cultural world (43, 45). It explored the role of looking at, drawing and talking about art objects as a source of mentalizing (28), principally through the role of joint attention (34, 39).

Each session lasted two hours and started with individuals sitting together around tables. Artwork made the previous week (drawing, paintings and clay models) was displayed on the wall or on chairs and formed part of the summary of the previous week. Individuals were asked to use their artwork and their reflections to remember the topic from the previous week. The topic for the week’s session was introduced with the addition of images projected onto the wall. For example, when exploring emotions of others, everyone looked together at portraits and reflected on differing interpretations of their emotions.

Co-therapists developed art exercises to help individuals learn and practice mentalizing skills, alongside taught material and handouts. An example exercise would be observational drawing to explore a ‘not knowing stance’. The principle of adopting a ‘not-knowing stance’ is key in mentalizing; the therapist maintains humility and an active and curious approach rather than making assumptions or interpretations about what is going on in the other person’s mind (37). In this exercise, individuals were asked to select a familiar object from their pocket or bag and draw it with curiosity as if seeing it for the first time. Drawing can been described as “*a marriage of what we know and what we see”* (41) and observational drawing is a helpful way of teasing out our assumptions from the curiosity involved in observation.

After a week or so individuals appeared comfortable to put their artwork up on the wall together. The transition from drawing in one’s own space to looking at the work with others is a significant and sometimes daunting shift, involving movement from one dimension to another [for instance, internal to external, as well as implicit to explicit (27, 30)]. Once the artwork was on the wall, individuals were asked to reflect on the exercise with reference to their art piece. They were encouraged to practice asking mentalizing questions of each other with the intention of stimulating each other’s’ mentalizing skills, as well as taking the opportunity to practice in a learning environment. Mentalizing questions can be simple curiosity that engages the individual and gets them to be curious about a particular aspect of the painting: “*What was it like to paint that bit?”* In comparison, apparent certainty and lack of curiosity might be something like: “*Wow that’s really good! You’re brilliant at drawing!”* A statement like this can sometimes be re-framed with the help of the therapist or others in the course: “*I really like your drawing, I’m trying to think what attracts me so much but I’m not sure what it is. Can you say a bit more about it?”*

Following the course, individuals were invited to share their reflections in Audio-Image Recordings (AIRs). AIRs have been developed by art therapists as a visual evaluation format and local examples are available to watch online (46). Individuals selected two to three art pieces to talk about in a recorded interview with a set template of questions. The interview was conducted with either therapist or another group member. Photos of the art pieces and the audio recording were edited together to make a short film which the individual kept as a record of their work. Some individuals gave consent for these recording to be used in education or research (47). Four individuals completed AIRs, and two provided co-therapists with further feedback on the course in a recorded focus group. Both the AIRs and the focus group were transcribed verbatim and compiled into an evaluation report that was presented locally within the trust. This evaluative project was registered and overseen by governance systems within the local NHS Trust. Although we do not refer to specific service user feedback in this paper (due to issues around consent), we have read and re-read these transcripts as part of our reflections on this course.

## 3 Experiential insights on delivering the Art MBT course

MBT practice is divided into a number of domains of intervention (37), some of which we have used here to organise our reflections. For the purposes of the Art MBT course we focused on the first four domains: ‘Not-knowing stance’, ‘Developing trust and structuring the sessions’, ‘Mentalizing process’ and ‘Pre-mentalizing modes’. Later domains, which focus on the relationship, sit within Art MBT therapy. A psychoeducational introduction, like the Art MBT course, needs to be in place to support the individual moving on to Art MBT therapy.

### 3.1 Not-knowing stance

Individuals on the course welcomed the notion of a curious, ‘not knowing’ stance, particularly about other people’s minds. They were interested to consider contrary evidence or alternative viewpoints, for example, reflecting on interpersonal interactions outside of the group in which they reported acting differently (or having wished to have done so) in terms of being curious about what was going on in their own mind and the mind of the other. This echoes experiences in other patient groups, where MBT courses have increased the ability to think with multiple perspectives (48). Within the studio we saw that individuals could both practice their not-knowing stance, and model it with each other.

This stance is particularly important on the part of the therapist, as commonly individuals who experience psychosis can feel unable to express their own thoughts and feelings, due to worries about how these will be perceived by others, or the fear of potential consequences [such as involuntary hospitalisation (49)]. We have discussed above how some individuals can struggle with feeling disconnected from their own thoughts, feelings and behaviours, necessitating a sensitive and non-judgmental approach by the therapist to help them start to connect with and sense their experience. Reflective curiosity can be particularly helpful in navigating the individual’s own personal experiences of psychosis (14). The medical co-therapist noticed that this felt like an important contrast to interactions where the clinician may feel it is their role to hold and impart knowledge.

### 3.2 Developing trust and structuring the session

Frequent reflections on uncertainty, paranoia and delusional ideas suggested significant experiences of fear and mistrust amongst group members. We also heard that experiencing psychosis can make it hard to be consoled by others, highlighting the fragility of attachment to others (50). Building epistemic trust needed particular attention and adaptions to practice, for example, making regular use of texts reminding individuals about the session, as well as give them a way to text back with information about late buses. More importantly, it explicitly modelled our intention to keep each individual in mind (18).

Springham and Camic underline how making art can invite feelings of failure if not carefully held by the therapist (31). Validating the art process and keeping a focus on it was important, as was working together at the same time. We notice a parallel with art education here, described as “*a drawing environment in which we can feel encouraged and supported, can dismantle our preconceptions, take risks, fail, make ‘bad’ drawings and allay our fears and inhibitions”* (41, p.9). This captures a quality of working with art materials that we suggest may help the individual to mentalize the self, in the company of the mentalizing group.

### 3.3 Mentalizing process

Individuals frequently linked heightened emotional arousal, stress and anxiety with a difficulty in expressing themselves or feeling blank-minded, reiterating the association between emotional dysregulation and poor mentalizing (14). Debbané refers to the therapist’s need to carefully scaffold interventions, from ‘safe’ to more activating components in order to move through the elements of MBT (14). An empathic and validating intervention (seeing things jointly from the individual’s perspective and taking care to understand their experience) is recognised as an essential first and ‘safe’ level of intervention to return to when emotional arousal is heightened, risking a rapid decrease in mentalizing. For example, if an individual was struggling to know how to start, we could pause the art making to ask others about their own experience. This was not so much to offer ideas and solutions (which often happened), but to share in the experience and validate it.

More activating interventions include using the MBT dimensions, for example, encouraging a individual stuck in a ‘cognitive state’ of overthinking to be curious about the ‘feeling’ in their art making, rebalancing the cognitive v affect dimension. In therapy (as distinct from the Art MBT course) the work develops to include the individual’s interpersonal relationship with the therapist and other group members and is placed at the deepest and most activating level in the spectrum of mentalizing interventions (37). It includes a focus on affective narrative, or the ‘elephant in the room’. This narrative might relate to deeply held feelings of mistrust or alienation that have not yet been approached or identified in therapy (14). Similarly, it may also be the place where the narrative of the individual’s avoidant attachment style can begin to be unpacked. The Art MBT course that is the subject of this paper is a foundation to this work up ahead.

We are interested in how using art in this process may have contributed to our ability to moderate the level of activation of our interventions and found that we became familiar with which materials represented safe-ground for different individuals. Soft dough, for example, was used in the first session with the intention of introducing individuals to art materials and to each other in a playful and easily achieved way. To describe this further: a structured exercise led individuals through a series of simple actions to form a body of dough with four legs and a tail from which point they could continue shaping the dough to create a real or imagined creature. The result tended to be a manageable experience involving touch, humour, playfulness and the production of something colourful and 3D. As described above, establishing a sense of safety and validation to return to was an important aspect of this course.

Individuals also created images relating to frightening psychotic experiences. We noticed when choosing images to speak about in the AIRs at the end of the course, it was often these images that were selected, suggesting some significance in the use of the art object to help make psychotic experience more explicit. Reflecting on this led us to develop a session devoted to the experience and definition of psychosis in future courses, called ‘What is Psychosis?’

Making and looking at art objects together offered a way of maintaining focus on the mentalizing task of the group. Moving from art-making individually to looking and talking about it together with others helped attendees question their understanding of what they had made, practicing shifts between internal and implicit processes to external and explicit ones, widely recognised as a mechanism of change (28, 30, 32).

Learning about some of the processes of MBT helped individuals step back and reflect on their experience, referred to by some as ‘giving distance’. Their understanding of implicit mentalizing was commonly adopted to describe making art and the artwork itself, while explicit expression was associated with both artwork and speaking together about it. ‘Pressing pause’, introduced as a way of pacing oneself and stopping to reflect on a situation, was also reported as something practiced at home. We saw the individual’s willingness to engage in new ideas as significant, underlining that opportunities to learn about mental health are important to this client group.

Co-therapists joined in the exercises with our own art making and reflections, identifying ourselves visibly as active participants in the group. As well as giving us the chance to self-mentalize, it invited opportunities for non-verbal dialogue between both therapists and group members. This could be used as a starting point to more explicit interactions, as thoughts and feeling were recognised and put into words (32, 35). In addition, having our images on the wall amongst others, invited individuals to be curious about what was going on in our minds as therapists, shifting our role to active participant rather than spectator or observer (32).

### 3.4 Pre-mentalizing modes

Individuals were able to grasp the essentials of mentalizing and referred to the usefulness of developing these abilities. They reflected on episodes of certainty that fears and delusions were real (associated with the pre-mentalizing state of psychotic equivalence), and were also able to identify these as a loss of mentalizing.

At times individuals described their reliance on the physical attributes of the art objects. For example, the use of art materials and their response to images was a way of finding expression when the individual felt that their ability to think was otherwise lacking or inaccessible. This use of the art object is associated elsewhere in MBT literature with teleological mode (29, 51). The physicality of the objects and the action may be particularly important when ‘thinking’ is difficult, while looking at them with others to understand some of their significance and meaning moves the process further towards a relational one.

On this first course we did not introduce the concept of pre-mentalizing modes into session material, although we have included them in subsequent courses. Once introduced, the pre-mentalizing ‘pretend mode’ and ‘psychic equivalence’ have been readily recognised by individuals on the courses and taken up as a way of naming experience.

## 4 Practice implications

Based on previous literature (14, 18) and our reflections, we recognise the importance of holding a non-judgmental, curious stance and building epistemic trust with those experiencing psychotic symptoms. Using a psychoeducational model within an MBT framework provided a clear structure to sessions. There was very good attendance, significant for a client group often associated with poor engagement. We noted that the inclusion of art encouraged most of the participants (but not all) to join the course, making it relatively accessible. We felt that more focus on pre-mentalizing modes was required, so that individuals could start to identify when they might be moving into one of these states. The balance of teaching, art making and looking together needs to be balanced, given that individuals vulnerable to psychosis may also find it hard to concentrate during the taught components of the course. Lastly, the expression of satisfaction and enjoyment in learning and making sense of personal experiences felt significant and resonates with previous literature highlighting the importance of self-agency and the experience of being seen and known (52).

## 5 Limitations

We are unable to make any assertions in this paper regarding individual feedback, nor wider implications of this work. The lag in time between the course and writing this paper means we are unable to seek further individual consent to share their feedback here. Our course had very small numbers and we were limited geographically to one locality within a trust, so generalisability is very limited. Moreover, during the process of writing this paper, the authors have developed the Art MBT service further to include an ongoing Art MBT therapy group, individual support sessions, as well as running several further Art MBT courses, which may have impacted on their reflections.

## 6 Future research

Future research into Art MBT for psychosis requires service user involvement from the start; this could include study design, retrieving feedback and presenting findings locally and within published literature. Qualitative exploration into group member experience of the Art MBT course would be highly valuable in understanding the potential processes of change in this new combined psychotherapy modality for psychosis. Research should also aim to take place with greater numbers of individuals and across more localities, so that generalisability is increased. Following this, if other teams decided to continue to a full Art MBT service (including group therapy and individual sessions), further research into the effectiveness of this service would be vital.

## 7 Conclusion

Combining art therapy and MBT offers an accessible and practical group structure within which to build trust, develop joint attention and increase mentalizing capacity. Adopting MBT’s ‘not knowing’ stance appeared to give group members more capacity to consider and discuss alternative viewpoints. A focus on attachment styles appeared very relevant and acceptable to individuals, often leading to exploration and discussion about social relationships with family and others, including trying to ‘understand misunderstandings’. We saw how mentalizing might operate within the use of art making in therapy in terms of the many different shifts demanded in its making and sharing. Art making may have further helped to slow down the mentalizing process to a manageable pace, making explicit mentalizing or expression of thoughts and feelings more possible. The enjoyment of art and creativity also made the course material more accessible for some. This Art MBT course introduces individuals experiencing FEP to a creative and social learning format in which they can practice joint attention and develop their mentalizing.

## Data availability statement

The original contributions presented in the study are included in the article/supplementary material. Further inquiries can be directed to the corresponding author.

## Author contributions

SP and CT were co therapists of the sessions described and wrote the first draft of the paper. SP and BC completed the second draft and the submitted version. All authors contributed to the article and approved the submitted version.
